# Purpureocillium lilacinum-related endophthalmitis: case report

**DOI:** 10.1186/s12348-024-00412-2

**Published:** 2024-06-12

**Authors:** Elena Ros-Sánchez, David Oliver-Gutierrez, Paul Buck, Tetiana Goncharova, Laura Distefano, Eric Kirkegaard-Biosca

**Affiliations:** Ophthalmology department, Vall d’Hebron University Hospital, Passeig de la Vall d’Hebron 129, Barcelona, 08035 Spain

**Keywords:** Endophthalmitis, Fungal endophthalmitis, *Purpureocillium lilacinum*, Evisceration, Case report

## Abstract

**Purpose:**

To report a case of *Purpureocillium lilacinum* endophthalmitis.

**Methods:**

The case of a fungal endophthalmitis caused by *Purpureocillium lilacinum* documented in an immunocompetent patient with no apparent trigger.

**Results:**

A 64-year-old male with a two-month history of panuveitis in his left eye was referred to our hospital. Initially misdiagnosed as sympathetic ophthalmia due to a previous surgery on his right eye 4 months before the onset of the left ocular picture, the patient received corticosteroid treatment, leading to a rapid deterioration of the left eye condition. An urgent exploratory vitrectomy was performed to identify the underlying cause, revealing endophthalmitis. Microbiological investigation yielded Purpureocillium lilacinum as the causative agent. Despite intensive treatment, including intravitreal antibiotics and antifungals, along with another surgical intervention, clinical evolution remained unfavourable, ultimately leading to the evisceration of the affected eye.

**Conclusions:**

*Purpureocillium lilacinum* poses a rare yet sever threat as a causative agent of fungal endophthalmitis. Managing such cases is challenging due to the delayed identification, fungus’s resistance to common antifungals, and its association with prior corticosteroid misuse in most patients. This case underscores the crucial importance of heightened clinical suspicion, early diagnosis, and the exploration of alternative treatment strategies in addressing *Purpureocillium lilacinum* endophthalmitis. The challenges posed by this rare fungal pathogen emphasize the need for a multidisciplinary approach and continued research to improve outcomes in these complex cases.

## Introduction

Endophthalmitis constitutes a sight-threatening condition that can result in profound visual impairment. Fungal endophthalmitis (FE) is notably less common than its bacterial counterpart, and its risk factors encompass immunosuppression, post-surgery and post-trauma conditions. Recognized culprits in FE include pathogens such as *Candida, Aspergillus* and *Fusarium*. However, less common agents also exist, capable of inflicting devastating prognosis in affected individuals [1, 2].

*Purpureocillium lilacinum (P. lilacinum)*, formerly identified as *Paecilomyces lilacinus*, is a globally distributed saprophytic filamentous fungus commonly present in soil and vegetation. While recognized for its role as a biological control agent against nematode pests, it can also act as an opportunistic pathogen, leading to severe infections such as endophthalmitis [3].

We present the case of a patient with a non-specific, two-month history of panuveitis, ultimately diagnosed with endophthalmitis caused by *P. lilacinum*, resulting in a devastating outcome.

## Case report

A 64-year-old male was referred to our hospital with a two-month history of panuveitis in his left eye. The patient’s medical history was arterial hypertension as sole pre-existing systemic condition. His ophthalmological background included high myopia. In his left eye, he had previously undergone cataract and vitrectomy surgery due to a rhegmatogenous retinal detachment a decade ago, with a final visual acuity of 0,4. In his right eye he underwent vitrectomy for rhegmatogenous retinal detachment four months prior to our visit.

During follow-up in another center, one month after the right eye vitrectomy, the left eye developed an inflammatory condition. The case was oriented as sympathetic ophthalmia (SO) and managed with topical and systemic corticosteroid treatment yielding no improvement. Consequently, one month after the beginning of the left eye ocular picture, the patient was referred to our hospital in order to initiate immunosuppressive therapy.

At first evaluation at our hospital, he was already undergoing treatment with oral prednisone (60 mg per day), methotrexate (20 mg per week), and prednisolone eye drops every 2 h. Visual acuity was counting fingers at 1 m in the right eye and light perception in the left eye. Right eye slit-lamp and fundus examination showed changes related to high myopia and previous history of retinal detachment surgery, with no other remarkable findings. Left eye presented corneal Descemet’s folds, + 4 cells in the anterior chamber and fine to intermediate keratic precipitates. Media opacity hindered fundus examination, and a B-scan ultrasound showed a marked hyperintensity primarily in the anterior vitreous cavity.

The working diagnosis remained unchanged. Pending baseline screening test results for the initiation of Adalimumab, a bridge therapy with an intravitreal dexamethasone implant was performed.

**Fig. 1:**
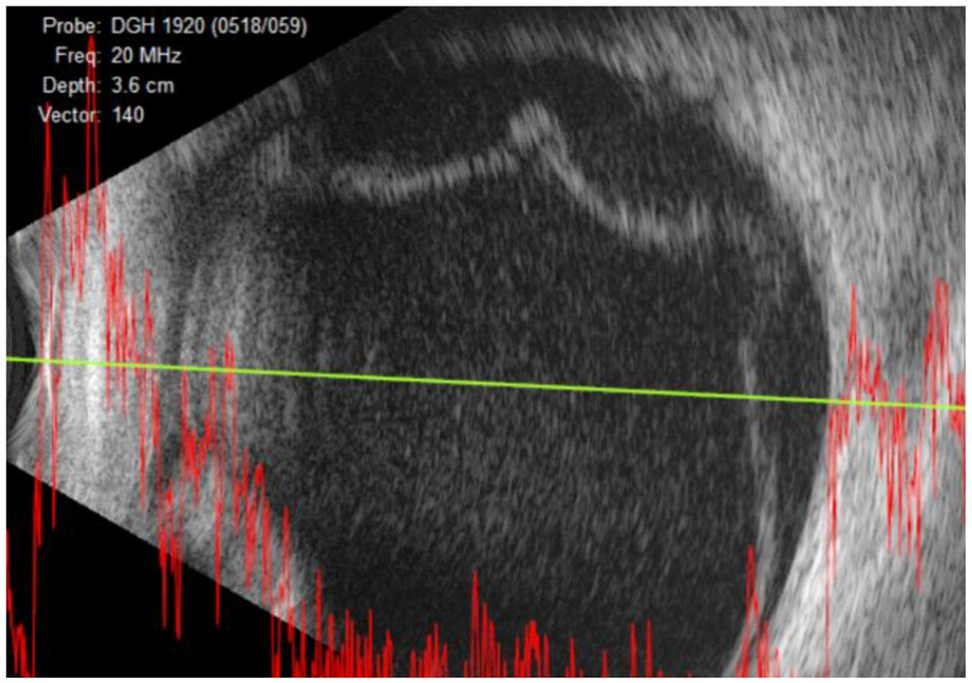
B-scan Ultrasound of the left eye six days after consultation at our hospital

Six days later, purulent retrolental material began depositing in the left eye. B-scan ultrasound revealed a hyperreflective image of lower density than the retina and intense echoes in the vitreous cavity (Fig. 1). Due to the deteriorating ophthalmological condition and endophthalmitis with retinal detachment suspicion, urgent exploratory vitrectomy was indicated.

The patient did not present any other clinical symptoms besides the ophthalmological mentioned. Additionally, blood cultures were collected, yielding negative results.

**Fig. 2:**
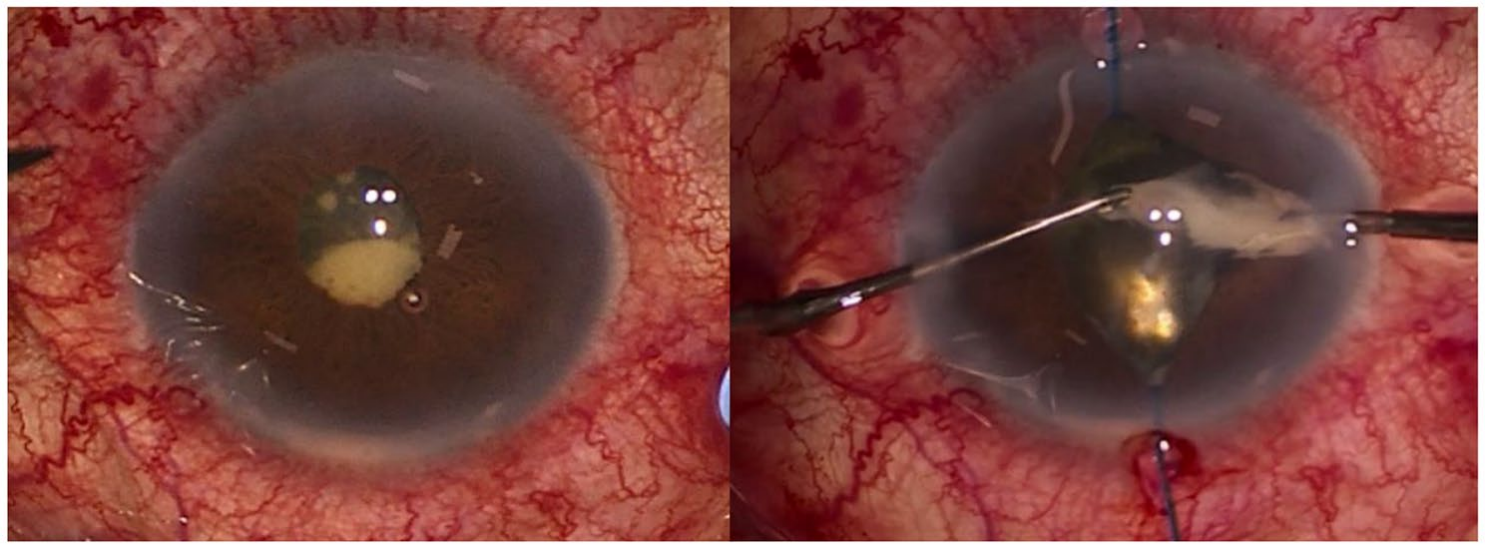
Left eye. At the beginning of surgery purulent material in the anterior chamber and retrolental region was extracted

**Fig. 3:**
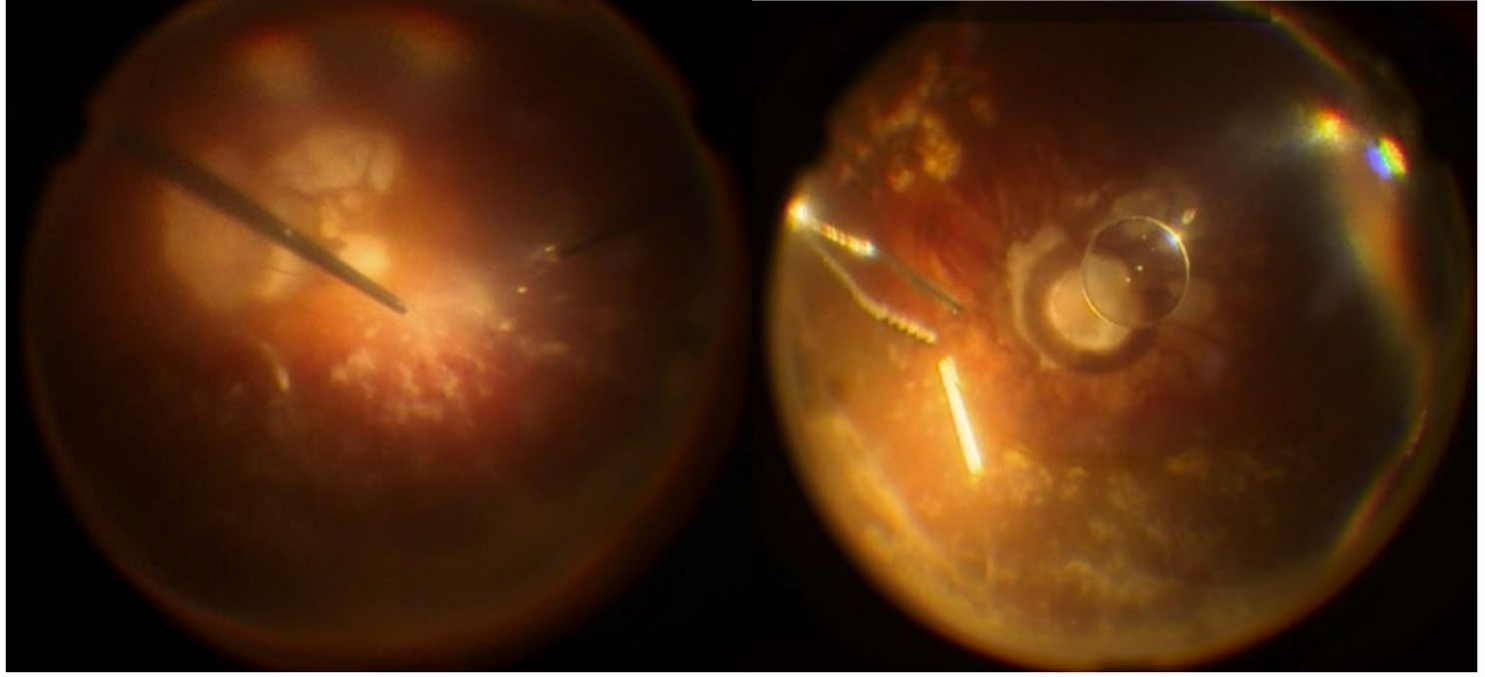
Left eye. Serous retinal detachment with preretinal haemorrhages and infiltrates

During the exploratory vitrectomy, purulent material in the anterior chamber and retrolental region was extracted and sent for microbiological analysis (Fig. 2). An inferior serous retinal detachment was observed, with preretinal infiltrates and microhaemorrhages on its surface (Fig. 3). The dexamethasone implant was removed and fluid was drained through a retinotomy, leaving silicone oil as a tamponade. Intravitreal injections of 0.1 ml of 2% ceftazidime and 1% vancomycin were administrated.

**Fig. 4:**
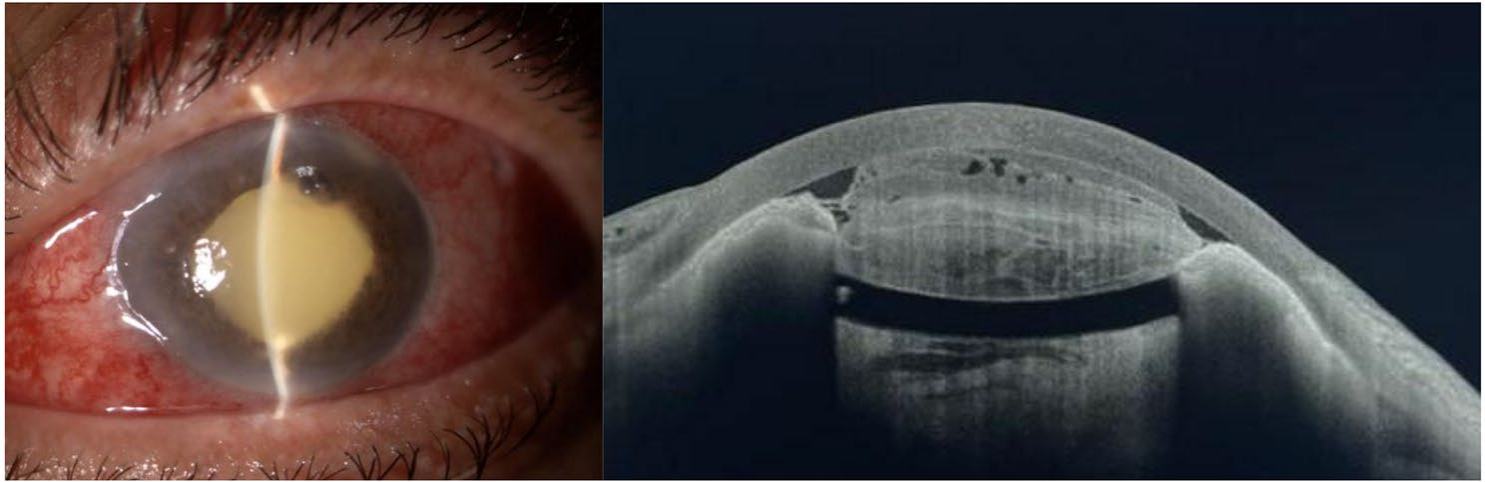
Left eye slit lamp examination and anterior segment OCT 7 days after first surgery

Two days after surgery, the left eye condition worsened, with pre and post-lens purulent material. On the fourth day post-surgery, + 4 cells and fibrinous deposits in the anterior chamber were observed. Microbiology results identified the growth of a filamentous fungus in vitreous cultures, prompting hospitalization and initiation of intravenous treatment and an intravitreal injection of 0.05 ml of 0.01% Amphotericin B deoxycholate. By the seventh day post-surgery, the condition had worsened despite changes on treatment, with hypotony and fibrinous and purulent deposits occupying over 50% of the anterior chamber. Vitreous cultures revealed the presence of *P. lilacinum (*Fig. 4*)*. Treatment was modified to intravenous Voriconazole (200 mg every 12 h) and intravitreal 0.05 ml of 0.05% Voriconazole.

**Fig. 5:**
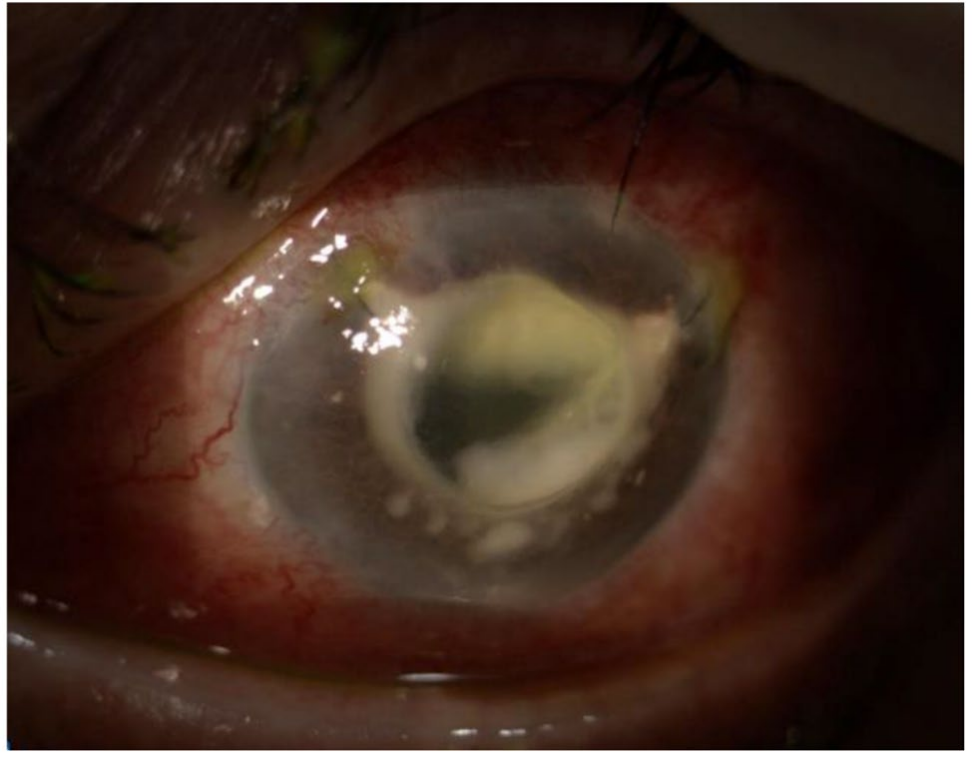
Left eye slit lamp examination after second surgery

Surgical anterior chamber debridement was performed, with intracamerular alteplase and Voriconazole injection, along with another intravitreal injection of Voriconazole. (Fig. 5). In the following two days, the left eye condition continued to worsen and the patient experienced sever ocular pain. An urgent evisceration was performed, and the excised eye structures were sent to microbiological and histopathological analysis.

The microbiological examination confirmed the presence of *P. lilacinum* in both corneal and intraocular samples. Histopathological analysis revealed necrotic and devitalized structures in the conjunctiva, cornea, and ciliary body, with positive staining results for Periodic Acid-Schiff (PAS) and Silver Methenamine, along with the presence of filamentous fungi. Retinal biopsy analysis disclosed necrotic fragments and fibrinoid material with areas of abscess formation.

## Discussion

*Purpureocillium lilacinum* is a saprophytic fungus that exhibits a particular affinity for ocular structures. Pastor et al. [4] conducted a comprehensive review of *P.lilacinum* infections, and among 119 reports spanning from 1964 to 2004, 51.3% corresponded to ocular mycosis. Key risk factors identified included intraocular lens implantation (32,8%), non-surgical trauma (20%) and ophthalmic surgery (10%). Notably, a significant number of patients had received corticosteroid treatment at the onset of ocular symptoms since these cases of endophthalmitis are frequently misdiagnosed. The outcomes revealed that 25% of cases experienced vision loss and 38% resulted in enucleation.

There is a limited number of reported cases of *P. lilacinum* in the literature. Guo et al. [5] reported a case of a 54-year-old immunocompetent woman initially misdiagnosed with uveitis, treated for two years with antibiotics and glucocorticoids. Upon suspicion of fungal endophthalmitis, she underwent two vitrectomies and antifungal therapy, achieving a final visual acuity of luminous perception. *P. lilacinum* was identified in surgical cultures. The patient had no significant medical history but had experienced a minor corneal injury two years before while caring for her grandchild, suggesting a potential entry point for the fungus and subsequent slow, progressive growth leading to chronic inflammation.

A recent case report highlighted *P. lilacinum* endophthalmitis in a 4-year-old immunocompetent child with no discernible risk factors. Initially misdiagnosed as panuveitis, the child’s visual acuity was ultimately diminished to no luminous perception despite surgery and antifungal treatment [6]. .

In line with the cases discussed, in our scenario, the patient exhibited no underlying systemic conditions and had not undergone immunosuppressive therapy prior the onset of the situation. Notably, there was no recent history of trauma to the left eye and the cataract and vitrectomy surgery were performed 10 years’ prior presentation.

The sole pertinent recent event was the surgery conducted on the fellow eye four months earlier, prompting suspicion of sympathetic ophthalmia (SO). Initial treatment for SO involves corticosteroids, while alternative immunomodulators and immunosuppresors have been traditionally contemplated for refractory and relapsing cases when tapering down corticosteroid treatment [7]. Nevertheless, for conditions such as SO, the early initiation of these medications has proven effective in improving long-term visual outcomes and minimizing ocular complications [8]. Jonas et al. documented a case of SO that did not respond to systemic steroids and immunosuppresors, and proposed intravitreal triamcinolone acetonide as an alternative treatment, as it proved successful in their case [9]. Regarding the anti-TNF therapy, Hiyama et al. reported two cases where Adalimumab demonstrated effectiveness as treatment for patients with a partial response to systemic corticosteroids and other immunosuppressors [10].

In our case, the left eye exhibited + 4 cells in anterior chamber despite treatment with 1 mg/kg/day of prednisone and 20 mg weekly methotrexate for nearly a month. Consequently, the decision was made to use intravitreal Dexamethasone as a bridge therapy before initiating Adalimumab. In hindsight, the unilateral nature of the ocular presentation and the absence of even a partial response to maximum corticosteroid treatment should have been considered as indicators that the condition was not primarily an inflammatory non-infectious process, as initially presumed. However, since no surgery, traumatic event or other systemic signs nor symptoms were evoked during extensive interrogation, infectious disease was not suspected.

An alternative hypothesis could be the onset of an exogenous fungal endophthalmitis following the intravitreal Dexamethasone implantation, indicated by the appearance of purulent material one week after the procedure. However, given the extended duration of ocular inflammation and the absence of response to previous treatments, the likelier scenario suggests fungal endophthalmitis from the beginning of the picture. Additionally, there are no documented cases of fungal endophthalmitis following intravitreal dexamethasone injection.

During the first surgery, purulent retrolental and vitreous material were extracted and sent for analysis. Vitreous cultures were conducted, revealing fungal growth four days post-surgery, with identification of *P. Lilacinum* one week after the procedure, delaying treatment modification and optimization. The implementation of KOH calcofluor white staining would have enabled direct visualization of the fungus and facilitated the initiation of antifungal treatment much sooner. Regrettably, obtaining this staining in our facility is exceedingly difficult.

Throughout the patient’s hospitalization, collaborative monitoring with the Infectious Diseases department revealed no other infectious focus that could account for endogenous endophthalmitis. After ruling out endogenous origins, the primary suspicion focused on an unnoticed microtrauma in a middle-aged patient or a gradually progressing infection, where inoculation might have occurred a decade prior during cataract and vitrectomy surgery. The likelihood of an undetected trauma seems minimal, as the patient did not report any symptoms such as conjunctival bleeding, abscess, trauma, or pain in the preceding years, and had not sought medical consultation. Conversely, it is acknowledged that fungal aetiologies can be insidious, often taking years to manifest. The presence of an inoculum in the lens could clarify the initial symptoms observed, with most of the inflammation predominantly located around the lens.

This case pose diagnostic challenges, as the absence of recent trauma, surgery or systemic condition can misleadingly exclude endophthalmitis from the differential diagnosis. The patient had been receiving systemic and topical corticosteroid treatment for the past two months, and during his stay at our hospital he underwent an intravitreal dexamethasone injection. This intervention may have exacerbated the situation by inducing local immunosuppression, thereby fostering the progression of the infection and the growth of the fungus, ultimately worsening the prognosis.

## Conclusions

Fungal endophthalmitis (FE), a rare yet devastating complication in ophthalmology, poses a significant challenge with the potential for severe visual impairment and blindness. The uncommon causative agent *P. Lilacinum*, adds complexity to the clinical landscape.

*P. Lilacinum* represents an exceedingly rare cause of FE, requiring consideration in patients with persistent inflammatory manifestations despite adequate treatment. Managing cases involving *P. Lilacinum* is inherently difficult due to its resistance to most antifungals, delayed identification, and prior corticosteroid use, contributing to a poor prognosis.

This cases underscores the importance of heightened clinical suspicion, early diagnosis, alternative therapeutic strategies, a multidisciplinary approach and ongoing research to enhance understanding and outcomes in such complex clinical scenarios.

## Data Availability

No datasets were generated or analysed during the current study.

## Abbreviations

FE: Fungal Endophthalmitis
P Lilacinum: Purpureocillium lilacinum
SO: Sympathetic Ophthalmia
